# Telehealth Use by Residence Type Among Older Adults Receiving Long-Term Services and Supports

**DOI:** 10.1093/geroni/igaf122.448

**Published:** 2025-12-31

**Authors:** Dana Urbanski, Romil Parikh, Jack Wolf, Benjamin Langworthy, Eric Jutkowitz, Tetyana Shippee

**Affiliations:** Indiana University Bloomington, Bloomington, Indiana, United States; University of Minnesota School of Public Health, Minneapolis, Minnesota, United States; University of Minnesota School of Public Health, Minneapolis, Minnesota, United States; University of Minnesota School of Public Health, Minneapolis, Minnesota, United States; University of Minnesota, Minneapolis, Minnesota, United States; University of Minnesota, Minneapolis, Minnesota, United States

## Abstract

Telehealth can improve access to geriatric care, particularly for vulnerable older adults facing transportation, mobility, and health-related barriers to in-person care. However, integration of telehealth into geriatric care must account for the different residence types where older adults use telehealth. This study examined the association between residence type and telehealth use among older recipients of long-term services and supports (LTSS) using cross-sectional data from the 2021-2022 National Core Indicators-Aging and Disability Adult Consumer Survey. The analytic sample included 6,925 LTSS users aged ≥ 65 years without intellectual or developmental disability: 69.2% lived in the community, 20% in residential care, and 10.9% in nursing facilities. Telehealth use was reported by 39.2% of community-dwelling respondents, 33.5% in residential care, and 20.3% in nursing facilities. Complete-case multivariable logistic regression, adjusting for sociodemographic and health-related factors with state-level random intercepts, revealed that those in residential care and nursing facilities had significantly lower adjusted odds of telehealth use than their community-dwelling counterparts (residential care: OR, 0.80 [95% CI, 0.69-0.92]; nursing facilities: OR, 0.37 [95% CI, 0.29-0.47]), with nursing home residents also having lower adjusted odds than those in residential care (OR, 0.46 [95% CI, 0.36-0.60]). These findings highlight differences in telehealth use among older LTSS users, with those in residential and nursing facilities having lower odds of telehealth use compared to community-dwelling individuals. Targeted efforts to promote telehealth adoption in institutional and congregate care settings may help bridge this gap.

